# Sensitive molecular detection of small nodal metastasis in uterine cervical cancer using HPV16-E6/CK19/MUC1 cancer biomarkers

**DOI:** 10.18632/oncotarget.24956

**Published:** 2018-04-24

**Authors:** Vanessa Samouëlian, Nawel Mechtouf, Eric Leblanc, Guillaume B. Cardin, Valérie Lhotellier, Denis Querleu, Françoise Révillion, Francis Rodier

**Affiliations:** ^1^ CRCHUM et Institut du cancer de Montréal, Montreal, QC, Canada; ^2^ Université de Montréal, Département d’Obstétrique Gynécologie, Montreal, QC, Canada; ^3^ CHUM, Service de Gynécologie oncologique, Montreal, QC, Canada; ^4^ Department of Surgery - Centre Oscar Lambret, Lille Cedex, France; ^5^ Laboratory of Human Molecular Oncology - Centre Oscar Lambret, Lille Cedex, France; ^6^ Institut Bergonie, Bordeaux, France; ^7^ Université de Montréal, Département de Radiologie, Radio-Oncologie et Médicine Nucléaire, Montreal, QC, Canada

**Keywords:** diagnostic of lymph node metastasis, HPV viral oncogenes, intraoperative pcr, pretherapeutic evaluation, RT-PCR, Pathology

## Abstract

Metastatic nodal involvement is a critical prognostic factor in uterine cervical cancer (UCC). To improve current methods of detecting UCC metastases in lymph nodes (LNs), we used quantitative PCR (qPCR) to assess mRNA expression of potential metastatic biomarkers. We found that expression of HPV16-E6, cytokeratin19 (CK19), and mucin1 (MUC1) is consistently upregulated in tumors and metastatic tissues, supporting a role for these genes in UCC progression. These putative biomarkers were able to predict the presence of histologically positive metastatic LNs with respective sensitivities and specificities of 82% and 99% (CK19), 76% and 95% (HPV16-E6), and 76% and 78% (MUC1). While the biomarkers failed to detect 1.7% to 2.2% of the histologically positive LNs when used individually, combining CK19 and HPV16-E6 enhanced sensitivity and specificity to 100% and 94%, respectively. To explore the sensitivity of qPCR-based detection of varying proportions of invading HPV16-positive UCC cells, we designed a LN metastasis model that achieved a fresh cell detection limit of 0.008% (1:12500 HPV16-positive to HPV16-negative cells), and a paraffin-embedded, formalin-fixed (PEFF) detection limit of 0.02% (1:5000 HPV16-positive to HPV16-negative cells), both of which are within the theoretical detection limit for micrometastasis. Thus, HPV E6/E7 oncogenes may be useful targets for the ultrasensitive detection of nodal involvements like micrometastases in fresh or archived tissue samples. Moreover, our results suggest that the biomarker combination of CK19/HPV-E6 could support a real-time intraoperative strategy for the detection of small, but potentially lethal, metastatic nodal involvements in fresh UCC tissues.

## INTRODUCTION

Uterine cervical cancer (UCC) is the fourth most common cancer and cause of death from cancer among women in the world (WORLD CANCER REPORT 2014 ISBN9283204298) [1]. UCC is triggered by human papillomavirus (HPV) infection. Among more than 200 characterized HPV subtypes [2, 3], at least 15 high-risk HPVs (HR-HPVs) are responsible for malignant progression [4–6]. The majority of cervical carcinomas are associated with the HR-HPV subtypes HPV16 and HPV18, which account for approximately 70% of UCCs worldwide [7]. The oncogenic potential of HR-HPV lies in the viral oncoproteins E6 and E7 [8], which bind and inactivate a number of essential tumor suppressors, leading to genomic instability and increased risk of malignant transformation in host cells [9–11].

Lymph node (LN) metastases comprised of epithelium-derived cervical cancer cells are a major negative prognostic factor in cervical carcinomas, and recent studies suggest that micrometastases are also predictive of tumor recurrence [12–14]. Whether the presence of smaller groups of isolated tumor cells (ITCs) is a risk factor remains controversial [13, 15, 16]. Hence, reliable assessment of LN metastasis and micrometastasis, including ITCs, becomes essential. The “gold standard” for nodal evaluation remains histological examination of 4 to 6 permanent sections assisted by immunohistochemistry (IHC) [17, 18]. However, in order to detect any micrometastatic involvement in a 1.5-cm LN, histological assessment requires 75 tissue sections. Time-consuming and expensive, this methodology is not suitable for the assessment of each node in everyday clinical practice, and a growing interest in the development of new methods to evaluate nodal involvement is emerging [14, 19, 20]. Several studies have shown that molecular analysis by real-time quantitative PCR (qPCR) is very efficient for the diagnosis of LN metastasis in other malignancies [21–23]. In cervical cancer, qPCR shows high sensitivity but low specificity for the detection of tumor cells in LNs [20, 24, 25].

In an attempt to improve the molecular detection of UCC nodal involvement, we evaluated the prognostic potential of several biomarkers that characterize the epithelial origin of cervical cancer cells, such as cytokeratin 19 (CK19), mucin 1 (MUC1), and human epidermal growth factor receptors 1-4 (HER 1-4) [26]. Since the HR-HPV oncogenes E6 and E7 induce the majority of cervical carcinomas, and are absolutely required for the initiation of UCC [4, 8], we also studied the expression of HPV16-E6/E7 as specific biomarkers of cervical uterine tumor cells. Lastly, we included the study of several proteins involved in cancer angiogenesis (vascular endothelial growth factor [VEGF], VEGF-C) and tumor cell invasion (urokinase-type plasminogen activator [uPA], matrix metalloproteinase 9 [MMP9]) to assess their predictive abilities as UCC metastatic biomarkers.

## RESULTS

### Detection of cancer biomarkers in normal cervical tissues, primary tumors, and lymph nodes

**Figure 1:**
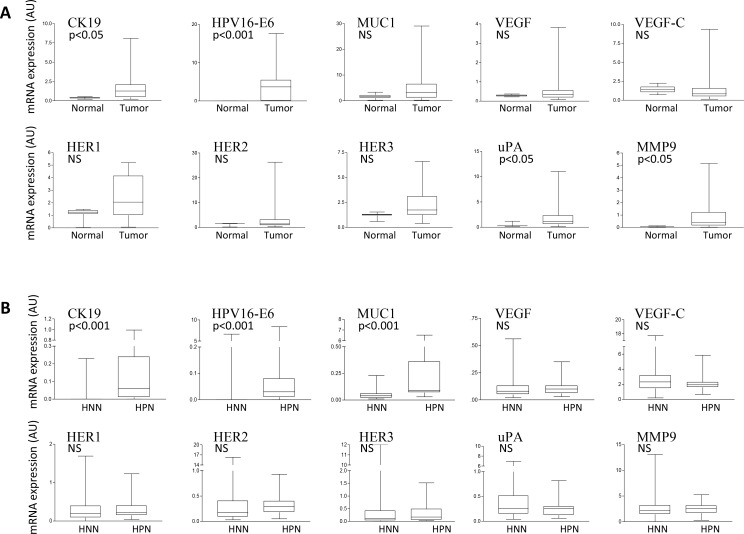
Cancer biomarker expression analysis in normal and UCC tissue samples (**A**) qPCR comparative gene expression analysis of the indicated cancer biomarkers in RNA from uterine normal cervix (Normal) or primary uterine cervical tumors (Tumor). The mRNA expression levels of each biomarker are reported in arbitrary units (AU) following internal normalization to TBP expression in the same sample. (**B**) RNA from histologically negative lymph nodes (HNNs) and histologically positive lymph nodes (HPNs) were analyzed as in a). NS: Not significant.

We first studied the RNA expression of 11 cancer biomarkers in normal uterine cervical and UCC tissues from patients with stage IB2–IVA cervical carcinoma. Four biomarkers had significantly higher expression levels in primary cervical tumors compared with normal cervical tissues: HPV16-E6 (*p <* 0.001), and CK19, uPA, and MMP9 (*p <* 0.05) (Figure 1A). Of note, HPV16-E6 was expressed in 15 of 21 primary cervical tumors (71.4%), but remained undetectable in all normal cervical tissues, as expected in UCC for this relatively common HR-HPV (Table 1). Among the 6 primary cervical tumors that did not express HPV16-E6, 2 were adenocarcinomas and 4 were squamous cell carcinomas (SCC). We then analyzed the expression of the cancer biomarkers in the 179 matching LNs of the 21 primary cervical tumors. When compared to histologically negative nodes (HNNs), we found that histologically positive nodes (HPNs) demonstrated a significantly higher expression of CK19, HPV16-E6, and MUC1 (*p <* 0.001) (Figure 1B). No conclusive observations were made regarding HER4, as this biomarker remained undetected in all types of samples analyzed.

**Table 1:** Assessment of histological and qPCR nodal involvement

LN/patient	HPN+	CK19	HPV16	MUC1	HER1	HER2	HER3	VEGF	VEGFC	uPA	MMP9
**3**	**3**	**3**	**1**	**2**	**1**	**1**	**1**	**2**	**2**	0	**1**
**7**	**2**	**3**	**2**	**5**	0	**1**	**2**	**1**	**3**	**2**	**1**
16^*^	**1**	**1**	0^*^	**2**	**4**	0	**4**	**4**	**4**	**2**	**1**
**15**	**7**	**6**	**9**	**9**	**3**	**5**	**4**	**6**	**5**	**3**	**6**
**9**	0	0	0	**4**	**5**	**6**	**4**	**5**	**3**	**1**	**1**
**8**	0	0	0	0	0	0	0	0	0	**1**	**1**
5^*^	0	0	0^*^	**1**	0	**1**	**1**	0	**4**	**4**	**1**
**11**	**2**	**1**	**4**	**4**	**11**	**7**	**5**	**8**	**9**	**8**	**2**
**4**	**2**	**2**	**2**	**2**	**2**	**1**	**2**	**1**	0	0	0
7^*^	0	0	0^*^	**4**	**4**	**4**	**3**	**4**	**2**	**3**	**5**
**6**	0	0	0	**2**	**2**	**4**	**3**	**1**	**1**	**3**	0
**6**	0	0	0	**5**	**5**	**4**	**3**	**2**	**1**	**1**	**1**
**7**	0	0	**1**	**1**	**1**	**2**	**1**	0	0	**1**	0
**8**	0	0	0	**2**	**4**	**4**	**3**	**5**	**5**	**3**	**3**
**12**	0	0	**2**	**6**	**1**	**2**	**2**	**1**	**1**	**1**	0
13^*^	0	0	0^*^	0	0	0	0	0	0	0	0
**8**	0	0	0	0	0	**1**	**3**	**1**	**1**	**1**	0
**11**	0	0	0	0	**2**	**1**	**2**	**1**	**1**	**1**	**2**
10^*^	0	0	0^*^	0	0	0	**1**	**1**	**2**	**1**	**10**
**7**	0	0	0	**3**	**1**	**1**	**2**	**1**	**1**	**4**	**4**
6^*^	0	0	0^*^	**1**	0	0	0	**2**	0	**2**	**5**

All individual LNs (*n* = 179) collected from the 21 patients included in this study were assessed for metastatic involvement using histology (HPN+) and the indicated qPCR molecular cancer biomarkers. The total number of LNs collected for each individual patient (LN/patient) is indicated in the first column, followed in each row by the number of LNs found positive for metastatic nodal involvement using the indicated assessment methods (histology or qPCR cancer biomarkers). ^*^Denotes patients with HPV16-E6–negative primary cervical tumors.

### Positive correlations between several cancer biomarkers

The expression levels of several independent cancer biomarkers were significantly correlated with each other in primary cervical tumors and HPNs. Specifically, 5 positive correlations were found in both primary cervical tumors and HPNs for the following pairs of genes: HER3–MUC1, HER3–uPA, HER1–HER2, HER2–HER3, and VEGF-C–MMP9 (Table 2). Uniquely in primary cervical tumors, we also observed 4 additional positively correlated gene pairs, including the HER family members HER1–HER3 and the invasion biomarkers uPA–MMP9. Finally, 5 additional positive correlations were found unique to HPNs; among these were the epithelial cell biomarker pairs CK19–MUC1 and CK19–HER3 (Table 2).

**Table 2:** Paired correlations for cancer biomarker expression levels in primary cervical tumors and HPNs (Spearman test)

Expression correlations in tumors			Expression correlation in HPNs		
Gene pairs	r	*p*	Gene pairs	r	*p*
MUC1 – HER3^*^	0.577	0.01	MUC1 – HER3^*^	0.679	0.003
HER1 – HER2^*^	0.752	<0.001	HER1 – HER2^*^	0.608	0.01
HER2 – HER3^*^	0.649	0.003	HER2 – HER3^*^	0.679	0.003
HER3 – uPA^*^	0.540	0.02	HER3 – uPA^*^	0.613	0.009
VEGF-C – MMP9^*^	0.525	0.02	VEGF-C – MMP9^*^	0.667	0.003
CK19 – HPV16-E6	0.498	0.03	CK19 – MUC1	0.828	0.001
HER1 – HER3	0.824	<0.001	CK19 – HER3	0.610	0.009
HER1 – uPA	0.473	0.04	HER1 – VEGF	0.586	0.013
MMP9 – uPA	0.639	0.003	HER2 – uPA	0.672	0.003
			uPA – VEGF-C	0.505	0.039

^*^Shared correlations between primary cervical tumors and HPNs.

### Evaluation of molecular identification of nodal involvement

To assess the predictive potential of the biomarkers to identify nodal involvement using qPCR, we first selected a cutoff value for the expression of each biomarker. The positive threshold of overexpression was chosen as the 75th percentile of the expression in the control HNN. Using this criterion, positive LNs according to each biomarker are listed in Table 1. Considering the 11 biomarkers separately, 13 to 53 LNs were identified to be positive (from a total of 17 HPNs). The sensitivity and specificity of the markers with significantly higher expression levels in HPNs compared with HNNs are reported in Table 3. As expected, we also observed that HPV16-E6 was never expressed in LNs from patients with HPV16-E6–negative primary cervical tumors.

**Table 3:** Agreement between molecular and histological assessment of LN involvement

A						
# LNs	Biomarker	qPCR–/H–	qPCR+/H+	qPCR+/H–	qPCR–/H+	% Agreement
179	CK19	160 (89%)	14 (8%)	2 (1%)	3 (2%)	97%
179	HPV16	153 (85%)	13 (7%)	9 (5%)	4 (2%)	92%
179	MUC1	122 (68%)	13 (7%)	40 (22%)	4 (2%)	75%
179	CK19 and HPV16 CK19 or HPV16	152 (85%)	10 (6%) 7 (4%)	1 (1%) 9 (5%)	0	95%

B						
# LNs	Biomarker	qPCR–/OHR–	qPCR+/OHR+	qPCR+/OHR–	qPCR–/OHR+	% Agreement
179	CK19	160 (89%)	15 (8%)	1 (1%)	3 (2%)	97%
179	HPV16	153 (85%)	16 (9%)	6 (3%)	4 (2%)	93%
179	MUC1	122 (68%)	16 (9%)	37 (21%)	4 (2%)	77%
179	CK19 and HPV16 CK19 or HPV16	152 (85%)	11 (6%) 10 (6%)	0 6 (3%)	0	97%

5a: Agreement between qPCR assay results and the original histological examination (H).

5b: Agreement between qPCR assay results and overall histological results (OHR).

H: Histological examination according to the standard procedures.

OHR: Overall histological result (histological examination according to the standard procedures combined with retrospective IHC for selected samples).

In each cell the data is presented as: Number of LNs (% out of 179 total LNs)

### Agreement between molecular evaluations and histological results

CK19 and HPV16-E6 detected a number of qPCR-positive nodes in the same range as the histological evaluation, 16 and 21 respectively (Table 1). Overall, qPCR agreement with the histological results was 97% for CK19, 92% for HPV16-E6, and 75% for MUC1 (Table 3A). The respective sensitivities (% of qPCR-positive nodes in the 17 HPNs) and specificities (% of qPCR-negative nodes in the 162 HNNs) for the molecular detection of histological nodal involvement were 82% and 99% for CK19, 76% and 95% for HPV16-E6, and 76% and 78% for MUC1. Each biomarker failed to detect about 2% (ranging from 1.7% to 2.2%) of the HPNs (Table 3A). Using a combination of CK19 and HPV16-E6, we observed a sensitivity of 100% and a specificity of 94%, reflecting a subset of 10 HNNs that were molecularly reclassified as positive (potential histological false negative or qPCR false positive). We thus performed a retrospective complementary histological examination in these 10 LNs and confirmed the presence of a micrometastasis in 1 sample and ITCs in 2 others. In summary, the final agreement between the combined CK19 and HPV16-E6 qPCR assessment and the revised overall histological result was 97% (Table 3B).

### UCC cell detection limits in complex samples using HPV16-E6/E7

**Figure 2:**
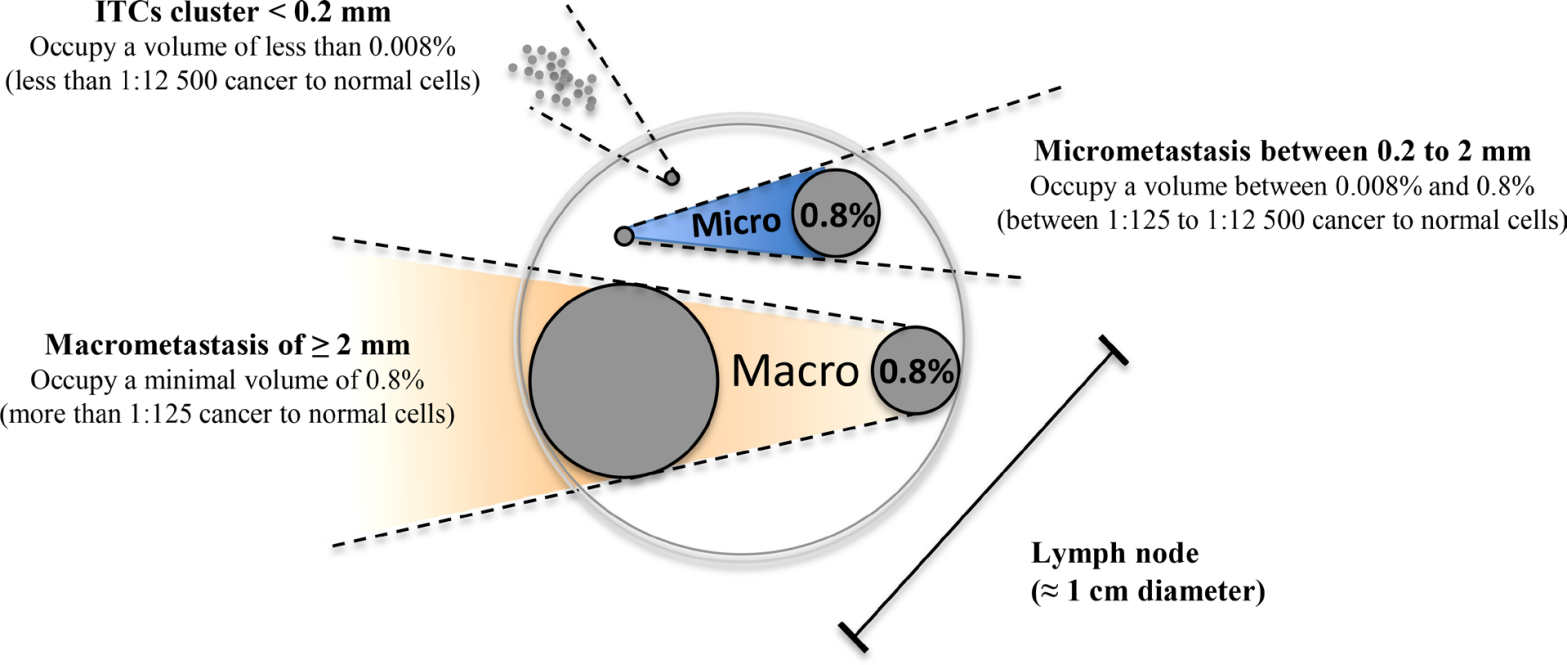
Modeled volume calculations for lymph node metastatic invasion Using mathematical modeling based on spherical volumes, it was determined that within a 1 cm lymph node, the transition between micrometastasis and macrometastasis occurs when the metastasis occupies 0.8% of the total volume of the lymph node (2 mm diameter). Structures larger than 0.8% of the total volume are considered macrometastasis (illustrated in orange), while structures between 0.8% and 0.008% are considered to be in the micrometastasis range (illustrated in blue). Isolated tumor cell (ITC) clusters occupy a volume of less than 0.008% of the lymph node.

**Figure 3:**
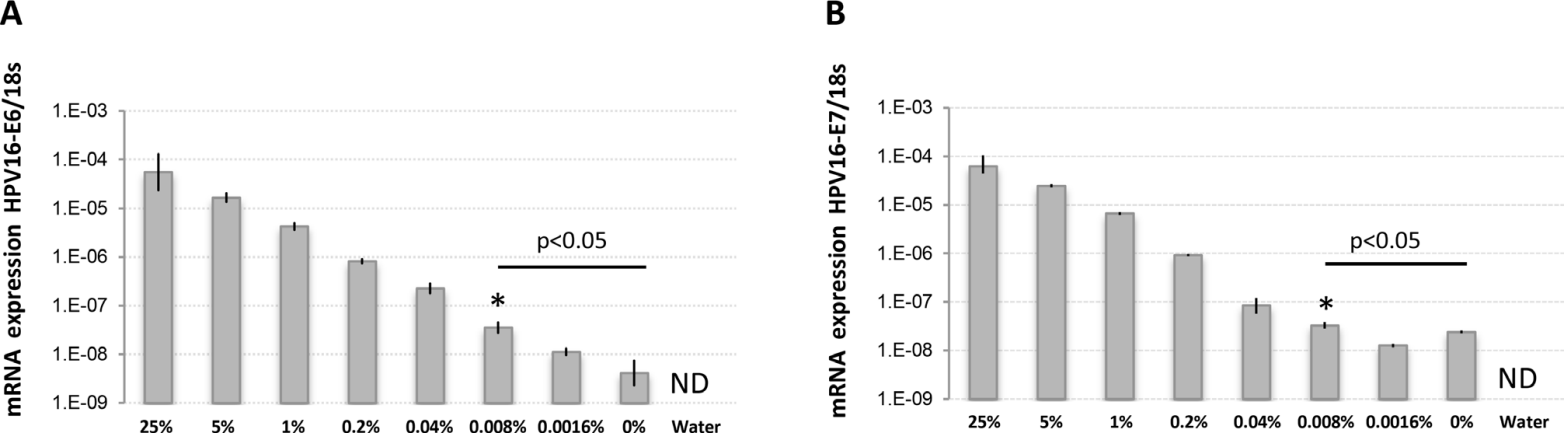
HPV16-E6 and HPV16-E7 detection limits in defined amounts of cancer cells using RNA extracted from fresh samples RNA was extracted from fresh populations of CaSki (HPV16-positive) and HCA2-hTERT (HPV16-negative) cells mixed at the indicated proportions and used for reverse-transcription and TaqMan qPCR. The percentage of CaSki cells in the sample is indicated. (**A**) Cell populations were analyzed for HPV16-E6 expression. (**B**) Cell populations were analyzed for HPV16-E7 expression. Note that the internal reference control, 18 s ribosomal RNA, was stably detected in all samples except water (not detected, ND), while HPV16-E6/E7 mRNA levels were gradually reduced according to CaSki cell dilutions. The data is presented as target mRNA expression relative to control (18 s RNA). A representative experiment with mean value ± SD of 3 replicates is presented. ^*^represents the highest dilution factor with statistical significance (*P <* 0.05, Students *T*-test) when compared to 0% dilution.

The high agreement rate found between histological and molecular detection suggest that LN involvement in UCC is at least equally detectable using either approaches. To quantitatively validate the ability of molecular biomarkers to detect trace UCC cells in complex tissue samples like metastatic LNs, we spiked defined amounts of live HPV16-positive UCC cells (CaSki) into HPV16-negative normal human diploid fibroblasts (HCA2-hTERT) in proportions that modeled various levels of infiltrating tumor cells. Normal pelvic LNs have an approximate upper limit size of 1 cm [27], while macrometastasis, micrometastasis, and ITCs are defined as tumor cell clusters with diameters of >2 mm, 0.2–2 mm, and <0.2 mm, respectively [28–30] We thus used a spherical volume evaluation formula (V = 4/3 × π × R^3^, where R = radius and π = 3.1415) to determine that macrometastases, micrometastases, and ITCs occupy >0.8%, 0.008–0.8%, and less than 0.008%, respectively, of the volume of an invaded LN (see model in Figure 2). RNA was then extracted from a sequential series of metastatic LN models, and the presence of HPV16-positive CaSki cells within otherwise HPV16-negative cellular samples was detected using qPCR. This method was able to detect HPV16-positive cell proportions as low as 0.008% of infiltrating cells (1 CaSki cell in 12500 total cells), which is well within the theoretical detection limit of micrometastasis (Figure 3).

### UCC cell detection limits in PEFF LN models using HPV16-E6/E7

**Figure 4:**
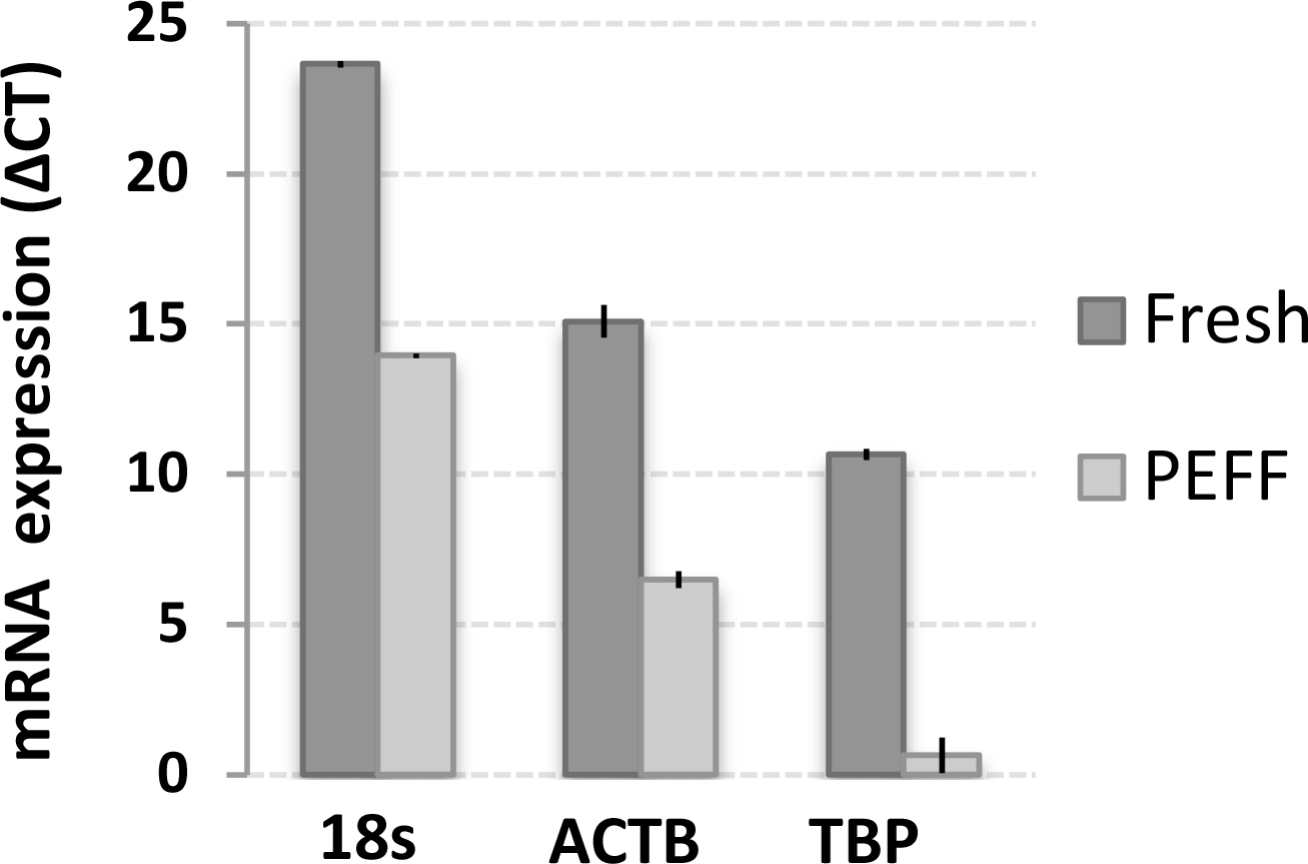
Differential sensitivity of gene expression detection in RNA extracted from matched fresh and PEFF samples RNA was extracted from matched samples of fresh or PEFF HeLa cells, and expression of beta actin (ACTB), TATA-binding protein (TBP) and 18s ribosomal RNA was analyzed using TaqMan qPCR. The data is presented as delta CT (cycle threshold), which illustrates the differences in PCR amplification cycles necessary to detect the various signals. A representative experiment with mean value ± SD of 3 replicates is presented.

**Figure 5:**
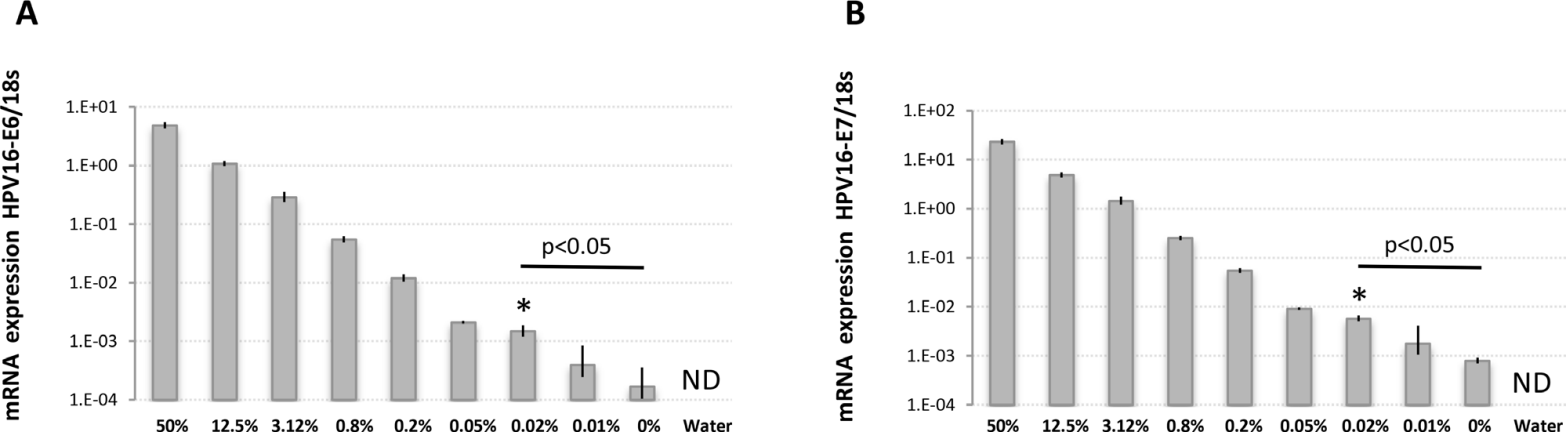
HPV16-E6 and HPV16-E7 detection limits in defined amounts of cancer cells using RNA extracted from PEFF samples RNA was extracted from PEFF populations of CaSki (HPV16-positive) and HCA2-hTERT (HPV16-negative) cells mixed at the indicated proportions and used for reverse-transcription and TaqMan qPCR. The percentage of CasKi cells in the sample is indicated. (**A**) Cell populations were analyzed for HPV16-E6 expression. (**B**) Cell populations were analyzed for HPV16-E7 expression. Note that the reference internal control, 18 s ribosomal RNA, was stably detected in all samples except water (not detected, ND), while HPV16-E6/E7 mRNA levels were gradually reduced according to CaSki cell dilutions. The data is presented as target mRNA expression relative to control (18 s RNA). A representative experiment with mean value ± SD of 3 replicates is presented. ^*^represents the highest dilution factor with statistical significance (*P <* 0.05, Students *T*-test) when compared to 0% dilution.

Archived paraffin-embedded, formalin-fixed PEFF tissue samples are the most common source of biological material from pathological assessment and are essential for retrospective studies. We thus evaluated the molecular detection limits in our metastatic LN invasion models that were prepared as above and immediately processed as PEFF. RNA was extracted from a series of PEFF metastatic LN samples, and the quality of the PEFF-RNA was compared to RNA extracted from fresh samples (Figure 4). Expression of the ribosomal 18s RNA, β-actin, and HPV16-E6 were easily measured, but TBP was barely detectable in PEFF samples. In fact, we observed a consistent reduction of about 10 amplification cycles in overall qPCR signals in PEFF samples, reflecting a roughly 1000-fold difference, most likely from PEFF-associated RNA degradation [31]. Using this validated PEFF-extracted RNA, the presence of HPV16-positive CaSki cells within otherwise HPV16-negative cellular samples was reliably detected down to a proportion of 0.02% infiltrating cells (1 CaSki cell in 5000 total cells), which is within the theoretical detection limit of micrometastasis (Figure 5).

## DISCUSSION

The aim of our study was to evaluate the use of qPCR biomarkers for the molecular detection of LN involvement in UCC. We investigated the expression of 11 genes selected for their importance as potential biomarkers of epithelial cells (CK19, MUC1, HER family), cervical uterine tumor cells (HPV16-E6), or for their involvement in tumor progression (uPA, MMP9, VEGF, VEGF-C).

### UCC biomarkers suitable for molecular detection of LN involvement

When comparing normal and tumoral uterine cervical tissues, we observed increased RNA expression of CK19, HVP16-E6, uPA, and MMP9 in the tumors, validating the importance of these genes in the UCC neoplastic process [32]. When comparing metastatic and non-metastatic LNs, we found that expression of CK19, HPV16-E6, and MUC1 was significantly higher in HPNs than HNNs (*p <* 0.001); therefore, these genes may be valuable candidates for the molecular detection of nodal invasion in UCC.

Importantly, HPV16-E6 had the lowest levels of non-specific background expression compared with the other biomarkers studied. Furthermore, it was undetectable in all normal cervical tissues and 6/21 of the UCC patient samples studied (corresponding to the 6 patients with HPV16-negative primary tumors; Table 1). This finding suggests that detection of HPV16-E6 could be a particularly sensitive strategy for the identification of HPV16-positive cancer cells in metastatic tissues. HPV16-E6 is an essential oncogene in HPV-induced cervical carcinogenesis, and is the reported causal agent of about 50% of UCCs worldwide [7], Interestingly, we found a higher proportion of HPV16-positive tumors in our cohort of patients (71%). Since HR-HPVs instigate more than 99% of UCCs, we expect that most of the 6 HPV16-E6–negative primary cervical tumors in our cohort were induced by a different HR-HPV subtype. Indeed, HPV16 is reported to be more frequently related to SCC, while HPV18 is commonly linked to adenocarcinomas [33], which could explain the absence of HPV16-E6 expression in at least 2 adenocarcinomas from our patient cohort. Given the high sensitivity and specificity of HPV16-E6 and HPV16-E7 as biomarkers of UCC nodal involvement achieved here, it would be interesting to develop molecular detection probes targeting E6/E7 oncoproteins from other HR-HPV subtypes.

Among our tested cancer biomarkers, only HER4 was not expressed in neither tumors nor LNs. In contrast to our results, previous studies have found increased HER4 expression in UCC tumors, particularly in patients with favorable outcomes [34, 35]. Similarly, HER4 suppresses breast cancer cell proliferation [36, 37], and the loss of HER4 expression during carcinogenesis contributes to the ability of breast tumor cells to avoid apoptosis [38]. Thus, it is possible that the lack of HER4 expression in the tumors that we have analyzed reflect their aggressive phenotypes, and perhaps HER4 would be detected in earlier stage tumors. Regardless, HER4 does not appear to be an appropriate molecular marker for the diagnosis of nodal involvement in UCC.

### Correlated expression of biomarkers reveals players in UCC metastatic invasion

When we compared the expression patterns of our 11 studied biomarkers, we observed significant associations between the co-expression of several markers of epithelial cells. For example, the expression of MUC1 and HER3 was correlated in both tumors and HPNs, while levels of MUC1 and CK19 were associated in HPNs. Thus, MUC1 and CK19 could be redundant biomarkers of nodal involvement. There was also a series of strong positive correlations among the detected HER family members (HER1 to 3) in the tumors as well as in HPNs. This result somewhat conflicts with a previously reported negative correlation between HER1 and HER2. However, expression in that study was evaluated at the protein level, perhaps explaining the differences observed with our RNA-based detection strategy [39].

The cancer invasion factor uPA was found to be positively correlated with numerous biomarkers in the tumor and/or HPN samples, including uPA, which is a known promoter of tumor invasion via the conversion of plasminogen to plasmin and the subsequent activation of procollagenases [32]. Our results suggest that uPA may similarly stimulate the invasion process in UCC, supporting a previously studied association between elevated uPA levels and the risk of LN metastasis in patients with poor prognoses [40]. Finally, we also observed correlations between VEGF-C and MMP9 in both tumors and HPNs, as previously reported [41]. Thus, alongside uPA, VEGF-C and MMP9 may also promote an invasive phenotype in UCC, potentially acting together to induce lymphatic spread.

### Using molecular biomarkers to detect rare invading UCC cells

When studying the concordance between classic histological assessment of LNs and the results obtained using qPCR molecular detection, we found that the highest performing biomarkers, CK19, HPV16-E6, and MUC1, still failed to detect about 2% of the HPNs when used individually. We thus moved forward to increase predictive ability by using a combination of two qPCR biomarkers, in hopes of matching the sensitivity and specificity seen with histological assessment. We chose CK19 and HPV16-E6 because they were the most significantly overexpressed biomarkers in HPNs, they had the best individual concordance with histological results (sensitivity and specificity), and importantly, their expressions were not correlated with each other in HPNs, indicating that they could provide complementary information. Indeed, using these 2 biomarkers together, 100% of HPNs were detected by molecular assessment. Moreover, an additional group of 10 HNNs was also found to be positive for CK19 and/or HPV16-E6, prompting us to perform a histological reassessment of these tissue samples using immunohistology. Reevaluation revealed confirmed metastatic involvement in 3 of the 10 cases (1 micrometastasis in 1 case and ITCs in 2 cases). Hence, as previously reported, and in support of our sensitivity assessments for the HR-HPV-E6/E7 biomarkers (see below), qPCR had a greater sensitivity to detect nodal involvement than routine histology. Unfortunately, the 7 remaining LNs in this group were not successfully re-assessed as positive by histology, suggesting qPCR false-positives, or metastatic nodal involvement present only in the portion of tissue used for RNA extraction. To ensure consistent tissue composition and to eliminate false-positive, at least for technique development, the next experimental validation step should be designed where consecutive sections of tissue are used for histological and molecular assessment, with slices sent to molecular assessment never being larger than the smallest size of the targeted metastatic lesion (i.e. to detect micrometastasis, an alternating allocation pattern of 150 µm-histology: 5 µm molecular assessment could be used). The sensitivity-effectiveness of detection should also be compared to the one-step nucleic acid amplification (OSNA) method, which was successfully used to detect UCC LNs metastatic involvement using CK19 as a single biomarker [42]. Overall, our results suggest that the combination of CK19 and HR-HPV-E6 identified in the primary tumor could be exploited in a qPCR or OSNA strategy for the detection of intraoperative nodal involvement with both high sensitivity and specificity in UCC patients. Positive results at that level could warrant the creation of a clinic-industry partnership to further develop this diagnostic strategy.

### Exploiting PEFF detection of rare invading UCC cells for retrospective studies

Using our cell culture spiking model, we were able to more precisely determine the ability of our qPCR molecular biomarkers to detect rare invading cancer cells in normal tissue. We demonstrated that HPV16-E6 and HPV16-E7 are both ultrasensitive molecular biomarkers for the accurate identification of HPV16-positive UCC cells in LN-like complex samples, with a detection limit of 0.008% cancer cells in fresh samples or 0.02% in PEFF samples. These results show that the qPCR detection strategy used here can reveal the presence of extremely small micrometastases, bordering the definition of ITCs, in fresh tissue samples, and can also detect micrometastases in PEFF samples. The applicability to PEFF samples is particularly important, as it suggests that qPCR detection of HPV-E6/E7 could be exploited to interrogate rich retrospective UCC tissue banks to definitively determine the importance of micrometastatic, and possibly ITC, involvement in UCC prognosis [28–30]. There is no doubt that the sensitivity of the technique can be improved, particularly in PEFF. Indeed, reducing the size of the qPCR target amplicons would mitigate the effect of the 1000-fold RNA degradation observed in these samples.

Overall, this study confirms that qPCR-based molecular detection of cancer biomarkers like CK19, HR-HPV-E6, and HR-HPV-E7 can be used to predict the presence of small nodal involvements, such as micrometastasis and perhaps even ITCs, in UCC. Given the near 100% involvement of HPV for this disease, a particularly efficient strategy would be to perform a pre-therapeutic HPV test that determines HPV subtype, which would be useful for intraoperative E6/E7 qPCR probes selection. Importantly, because HR-HPV is known to cause a high fraction of anal (90%), subsets of oropharyngeal (60%), vaginal (40%), vulval (40%), and penile (40%) cancers [43], the biomarker combinations proposed here could potentially be extended to an even wider prognostic use.

## MATERIALS AND METHODS

### Patients

All patients signed informed consent forms as part of an ethical protocol approved by the Centre Oscar Lambret (Lille). Uterine cervical tumor and LN samples were collected from 21 patients with cervical carcinoma (stages IB2-IVA). Patients with locally advanced UCC and no enlarged aortic nodes observed through magnetic resonance imaging (MRI) systematically underwent a surgical pretherapeutic nodal assessment. The surgical staging in this group of patients included a diagnostic transperitoneal laparoscopic abdominopelvic exploration to preclude patients with obvious peritoneal carcinomatosis and/or fixed pelvic or paraaortic LNs. If negative, a retroperitoneal laparoscopic infrarenal paraaortic LN dissection was performed as described [44]. A biopsy of the uterine cervical tumor was performed at the time of surgery. The tumor biopsies were cut in half for histological evaluation and molecular assessment. LNs that were greater than 0.5 mm were cut in half. One half was analyzed using conventional histological examination, while the second half was snap frozen and stored in liquid nitrogen until RNA extraction. LNs that were smaller than 0.5 mm were examined in anatomopathology only (thus not included in this study). Among the 179 LNs assessed by histology and qPCR, 17 were histologically positive. Normal cervical tissue was collected from patients with normal Pap tests who had a vaginal hysterectomy, for benign uterine pathology.

The median age of the 21 surgically staged patients was 43 years (26 to 56 years). Nine patients presented stage IB2 primary uterine cervical carcinoma, 9 had stage IIB, and 3 had stage IIIA. Eighteen women had histologically confirmed uterine cervical SCC, 2 had adenocarcinomas, and 1 had an adenosquamous carcinoma. The average number of paraaortic nodes was 31 per patient (range: 17–43). Six patients had histologically confirmed paraaortic nodal involvement with at least 1 macrometastase (≥ 2 mm), and 4 patients had both macro and micrometastases (≥ 0.2mm and < 2 mm). The median follow-up was 36 months (27 to 40 months). Seven patients developed a recurrence after a median of 12 months (3–35 months). Three of these patients had positive paraaortic LNs. Recurrence was locoregional in 5 cases. One patient had locoregional and distant recurrence.

### Cell lines

Cells were purchased from the American Type Culture Collection. MCF7, T47D, and MDA-MB-231 cells were cultured in MEM, HeLa in DMEM, and CaSki in RPMI. All culture media were supplemented with 10% fetal calf serum, 100 IU/ml penicillin, and 100 µg/ml streptomycin, with 2 mM glutamine also added to the MEM media. The cells were grown at 37° C in a humidified atmosphere of 5% CO_2_, and were collected at sub-confluence.

### Histological examination

LNs were cut in 2-to-3 mm tissue slices and then paraffin-embedded and formalin-fixed (PEFF). For each block, three 5-µm-thick full sections were collected at 3 levels, 300 µm apart. Hematoxylin/eosin (H&E) stained sections were used for histological examination. Confirmatory IHC was performed on request from the pathologist using anti-keratin antibodies AE1/AE3. Histologically negative LNs detected as positive by qPCR were also similarly reexamined using IHC.

### RNA extraction

Fresh tumors and LNs samples were frozen and stored in liquid nitrogen until RNA extraction. Total RNA was isolated using the MagNa Pure Compact system (Roche Applied Science) with the RNA Isolation From Human Tissue Kit (Roche Applied Science). Disruption and homogenization of tissue samples were performed using a Rotor-Stator Homogenizer (Ribolyzer, Hybaid). For modeled micrometastases, total RNA from CaSki and HeLa cell lines was isolated using TRIzol^®^ (Invitrogen) following the manufacturer’s instructions. The purity of the RNA was verified with the 260/280 nm ratio and confirmed by electrophoresis.

For PEFF samples, RNA extraction was done using the RecoverAll™ Total Nucleic Acid Isolation Kit (Ambion, Inc.) with minor changes to the manufacturer’s protocol. Briefly, fifteen 20-µm-thick paraffin sections from each PEFF sample were used per extraction, and the proteinase digestion was lengthened to 1 hour at 50° C and extended for another 15 minutes if the sample was still viscous. This was repeated as needed until the sample became clear. The resulting RNA eluate was repurified with the RNeasy^**®**^ MinElute™ Cleanup Kit (QIAGEN).

### PCR primers and TaqMan fluorogenic probe

The sequences of the primers and the TaqMan fluorogenic probes are presented in Table 4. In order to confirm the total gene specificity of the sequences chosen for the primers and probes, we performed BLASTn searches against dbEST and nr (the non-redundant set of GenBank, EMBL, and DDBJ database sequences). Primers and probes were purchased from Applied Biosystems (Courtabœuf, France), Proligo (Saint Quentin Fallavier, France), and Integrated DNA (Coralville, USA).

**Table 4:** Sequences of primers and TaqMan fluorogenic probes

Gene		Sequence (5′-3′)	Product size (bp)
**r18s**			
Forward Primer	cggctaccacatccaaggaa		197
Reverse Primer	cgctattggagctggaatta		
Probe		(HEX) – tgctggcaccagacttgccctc– (BHQ-1)	
**CK19**			
Forward Primer	tcgacaacgcccgtctg		75
Reverse Primer	ccacgctcatgcgcag		
Probe		(6FAM) – cctgttccgtctcaaacttggttcgg – (TAMRA)	
**HER1**			
Forward Primer	tccccgtaattatgtggtgacagatc		250
Reverse Primer	acccctaaatgccaccggc		
Probe		(6FAM) – cagctatgagatggaggaagacggcgt – (TAMRA)	
**HER2**			
Forward Primer	caaccaagtgaggcaggtcc		101
Reverse Primer	ggtctccattgtctagcacgg		
Probe		(6FAM) – agaggctgcggattgtgcga – (TAMRA)	
**HER3**			
Forward Primer	gggagccgcttccagact		98
Reverse Primer	ttgaggccggtgatcagaaa		
Probe		(6FAM) – tggactcgagcaacattgatggatttgt – (TAMRA)	
**HER4**			
Forward Primer	tgttcggaacccatggcct		167
Reverse Primer	agcatctgccgtcacattgttct		
Probe		(6FAM) – atggtagttcaggatgtggacgttgcca – (TAMRA)	
**MMP9**			
Forward Primer	ccctggagacctgagaacca		77
Reverse Primer	cccgagtgtaaccatagcgg		
Probe		(6FAM) – attcctctgccagctgcctgtcg – (TAMRA)	
**Muc-1**			
Forward Primer	gtgccccctagcagtaccg		123
Reverse Primer	gacgtgcccctacaagttgg		
Probe		(6FAM) – cattacctgcagaaaccttctcataggggct – (TAMRA)	
**HPV16-E6**			
Forward Primer	aatgtttcaggacccacagg		124
Reverse Primer	ctcacgtcgcagtaactgttg		
Probe		(6FAM) – cgacccagaaagtt – (TAMRA)	
**HPV16-E7**			
Forward Primer	caagcagaaccggacaga		96
Reverse Primer	gtctacgtgtgtgctttgta		
Probe		(ROX) – caagtgtgactctacgcttcggttgtg – (BHQ-2)	
**TBP**			
Forward Primer	cacgaaccacggcactgatt		89
Reverse Primer	ttttcttgctgccagtctggac		
Probe		(VIC) – tcttcactcttggctcctgtgcaca – (TAMRA)	
**uPA**			
Forward Primer	actgcaggaacccagacaacc		70
Reverse Primer	tggacaagcggctttaggc		
Probe		(6FAM) – ctgcacatagcaccagggtcgcct – (TAMRA)	
**VEGF-C**			
Forward Primer	tcaaggacagaagagactataaaatttgc		137
Reverse Primer	actccaaactccttccccacat		
Probe		(6FAM) – atacacacctcccgtggcatgcattg – (TAMRA)	
**VEGF**			
Forward Primer	gcacccatggcagaagg		90
Reverse Primer	ctcgattggatggcagtagct		
Probe		(6FAM) – ctgatagacatccatgaacttcaccacttcgt – (TAMRA)	

### TaqMan qPCR

We studied the expression of CK19, HPV16-E6, MUC1, uPA, HER1 (EGFR), HER2, HER3, HER4, MMP9, VEGF, and VEGF-C in normal uterine tissues, uterine cervical tumors, and in paraaortic LNs using the housekeeping gene TBP (TATA box Binding Protein) as a normalizing reference [45]. Negative and positive controls were included in each experiment, using cell lines known to express each gene as positive controls. The non-template controls and the samples were assayed in duplicate. Actin beta (ACTB), Ribosomal 18 s (r18s), and HPV16-E6/E7 were analyzed in samples from UCC cell lines (CaSki and HeLa).

### qPCR conditions

For primary tissues, RNA-DNA reverse transcription and qPCR were performed in a one-step methodology on 50 ng of total RNA using a 7700 ABI PRISM sequence detector system (Applied Biosystems). The reaction mixture (20 µl final volume) contained 2X Master Mix (Eurogentec, Seraing, Belgique), including HotGold Star DNA polymerase, dNTP and MgCl_2,_ 5 units RNase inhibitor, 2 units Euroscript Reverse transcriptase, primers, and probe (Proligo, France) at the concentration indicated in Table 5. Reverse transcription was performed at 48° C for 30 min. The activation of the DNA polymerase (10 min at 95° C) was followed by PCR (15 sec at 95° C, 1 min at 60° C [except CK19, which was performed for 1 min at 62° C], and 1 min at 72° C for 40 cycles). For PEFF samples, the RNA-DNA reverse transcription was done using the SuperScript^**®**^ III First-Strand Synthesis SuperMix for qRT-PCR kit (Invitrogen) with 1 µg template RNA, and the qPCR was performed on the Rotor-Gene RG-3000 (Corbett Research) with the Rotor Gene 6 software, using the platinum qPCR SuperMix-UDG kit (Invitrogen) and 15 µL reactions.

**Table 5:** Concentrations used for primers and TaqMan fluorogenic probes

Gene	Primers (nM)	Probe (nM)
CK19	400	200
HPV16-E6, HPV-E7	200	200
MUC1	200	300
HER1-4, TBP, r18s	200	200
uPA	300	300
VEGF, VEGF-C	400	200
MMP9	400	400

### Relative quantification of target gene expression

The relative quantification of each target gene expression was performed using the comparative cycle threshold (C_T_) method, where the C_T_ parameter is defined as the cycle number at which the fluorescent signal generated by cleavage of the dual labeled probe is first detectable. This method is based upon the use of a calibrator sample (i.e. 1× sample), which allows for quantification in the unknown samples. We used pooled uterine cervix cancer tumors as calibrator (i.e. target expression = 1). The relative target expression was calculated with the formula, 2^–ΔΔCt^, where ΔΔC_T_= ΔC_T_ patient sample –ΔC_T_ calibrator sample, with ΔC_T_ = C_T__TARGET_ – C_T__TBP_.

### Statistical analyses

Correlations between the biomarkers were assessed according to the Spearman test. All statistical analyses were done using the SPSS software (Version 13.0.1). Nonparametric Mann–Whitney and Kruskal–Wallis tests were used to compare expressions of the target genes in tumors and LNs.
